# How to utilise your time effectively during the Covid-19 pandemic

**DOI:** 10.15694/mep.2020.000115.1

**Published:** 2020-05-28

**Authors:** Daniyal Raja

**Affiliations:** 1 University of Exeter

**Keywords:** Covid-19, medical student, education, future planning, mental health

## Abstract

This article was migrated. The article was marked as recommended.

United Kingdom (UK) medical schools have decided to temporarily discontinue face-to-face teaching and clinical placements, due to the Covid-19 pandemic. This has meant that medical students across the country are to study remotely and having no exams for a long period of time can seem strange to a group of people who are continuously assessed. As a current fourth-year medical student at the University of Exeter and a future medical practitioner, it is nevertheless important to appreciate that doctors have a duty of keeping their knowledge and skills up to date (GMC, 2019). This article will highlight the key ways medical students can spend their time productively during lockdown.

## Working in isolation

Studying medicine requires constant learning and it is imperative to use techniques that work well for you. Medical students tend to be multimodal in their learning styles, which means they are versatile with the techniques they use to grasp new information ( Prithishkumar and Michael, 2014). Although the current circumstances are disruptive, this is the ideal time to explore new learning strategies that support long-term knowledge retention and there are a numerous ways that this can be achieved. Examples include:

• Hand-written notes - Note-taking from written and online teaching materials can help consolidate learning and allows you to revisit topics in the future.
• Flashcards - Flashcards can be created to summarise topics, such as, physiology or pharmacology, and are easy to transport if you study in different areas of the home. If preferable, online versions can be used and shared electronically to fellow peers.
• Mind maps - Visual representation through mind maps simplifies complex material by breaking down information into smaller chunks.
• Mnemonics - Mnemonics are effective, because they associate new information with knowledge that is already stored in your long-term memory.
• Question banks - Using question banks, such as Passmedicine ( Passmedicine, 2020) will widen your scope on pathology and help you prepare for future exams.

Utilisation of spaced repetition and testing effect (answering questions to strengthen memory) will help retain knowledge over longer periods. Flashcards and question banks in particular, enable you to implement these learning theories.

Interaction with others is also a key element of medical education. While limited exposure to the public makes contact with others difficult, it is not impossible. Organising video calls with a group of peers to discuss clinical cases allows you to build on existing knowledge and learn from one another. Using a Problem-Based Learning (PBL) format will help boost clinical acumen, as you have the opportunity to approach cases as a clinician, simultaneously enhancing your interpersonal and teamworking skills. Medical schools that use the PBL approach will have cases available on their e-learning environments, however sample cases can also be found online.

Some Universities are also providing online lectures and tutorials. Attending these will ensure that you cover the topics included in your medical school curriculum. You will also likely have the chance to ask questions to the clinician or expert delivering the lecture, which is an invaluable opportunity often taken for granted.

## Planning for the future

Devote the spare time you have to plan for the future and explore different aspects of medicine. A few hours of basic research on different medical and surgical specialties may help you develop an area of interest. More senior medical students can avoid procrastinating by gaining a better understanding of the UK foundation programme, different training pathways and start planning for electives.

It is worth utilising platforms, such as Microsoft Teams, to develop your teaching skills. Doctors are expected to contribute to teaching during their careers and be good role models to other members of the team. The word ‘doctor’ itself is derived from the Latin term ‘docere’, which means ‘to teach’ ( Lawson and Ling, 2004). Delivering online lectures on topics that are of interest or not covered in depth by medical schools can benefit junior students. Ensure that you collect electronic feedback forms, as this will validate your commitment to teaching and enhance your portfolio. Other options include mentoring or providing online tuition to school children. This form of teaching would benefit students and parents alike during school closures, whilst also providing a means of income.

In addition to teaching, winning prize essay competitions and obtaining publications are effective ways to develop your portfolio and prepare for future academic work. Submitting articles to student-run journals is a good place to start if you are new to research and writing articles.

## Mental health and well-being

Having minimal human contact and making unusual changes to your regular norms can be stressful. Make sure to invest time in the well-being of yourself and others in your local community. Check in on family members and elderly neighbours via phone or video call, especially if they live alone. In addition to hospital volunteering, offering to help NHS workers with errands, such as, shopping or pet care can be of huge benefit. However, only volunteer if you are well enough and are not in a high-risk group.

To support your own mental health, practising good self-care is paramount. It is easy to consume unhealthy foods and become lazy. A daily routine is crucial to maintain a healthy diet, good sleep pattern and personal hygiene. Do regular exercise, even if this is merely a short walk outdoors (sticking to social distancing measures).

At present, the exact course of the Covid-19 pandemic is unpredictable. Until Universities resume typical custom, medical students should continue to educate themselves remotely and dedicate time to their future development, whilst also supporting the well-being of themselves and others.

## Take Home Messages

• The Covid-19 pandemic has meant that medical education must be delivered remotely
• This is the ideal time for medical students to explore new learning strategies
• Medical students should continue to work with their peers, using online learning platforms
• Medical students should allocate time to plan for their careers and future
• Medical students should prioritise their mental health and the well-being of others

## Notes On Contributors

Daniyal Raja is a medical student at the University of Exeter. ORCID ID: https://orcid.org/0000-0002-6434-764X
