# Supplementary material for: How to utilise your time effectively during the Covid-19 pandemic
Source: MedEdPublish (2016). 2020 May 28;9:115. [Version 1] doi: 10.15694/mep.2020.000115.1 (PMC10702640; doi:10.15694/mep.2020.000115.1)
Supplement: Peer review history

# Reviewer report: Reviewer response for version 1

**DOI:** 10.21956/mep.20092.r30949

**Published:** 2020-06-11

**Authors:** Wafaa Mowlabaccus

**Affiliations:** 1 University of Mauritius

This review has been migrated. The reviewer awarded 4 stars out of 5 Being a medical student, I find this article very useful and comprehensive. The author managed to suggest cost effective and simple ways to make good use of the time during a pandemic.

Reviewer Expertise:

NA

No decision status is available

# Reviewer report: Reviewer response for version 1

**DOI:** 10.21956/mep.20092.r30950

**Published:** 2020-06-11

**Authors:** Hebat Allah A. Amin

**Affiliations:** 1 Department of Pathology, Helwan University, Faculty of Medicine

This review has been migrated. The reviewer awarded 5 stars out of 5 Excellent guide from a medical school student. I do recommend it and will share it with my students. Authentic work, much appreciated.

Reviewer Expertise:

NA

No decision status is available

# Reviewer report: Reviewer response for version 1

**DOI:** 10.21956/mep.20092.r30951

**Published:** 2020-06-11

**Authors:** Nagwa Hegazy

**Affiliations:** 1 Menoufia university

This review has been migrated. The reviewer awarded 4 stars out of 5 Great advises specifically working in isolation. Thanks

Reviewer Expertise:

NA

No decision status is available

# Reviewer report: Reviewer response for version 1

**DOI:** 10.21956/mep.20092.r30947

**Published:** 2020-06-10

**Authors:** Susmita Reddy Karri

**Affiliations:** 1 Topiwala national medical college and BYL nair charitable hospital, mumbai. india

This review has been migrated. The reviewer awarded 4 stars out of 5 A very well structured article. What I really like is the focus on mental health- something that we often forget! Statistics show that we lose a doctor every 3 weeks to mental health issues. In this COVID era, I believe that it is all the more important to make sure that or colleagues, including medical students have help and support. Great job!

Reviewer Expertise:

NA

No decision status is available

# Reviewer report: Reviewer response for version 1

**DOI:** 10.21956/mep.20092.r30948

**Published:** 2020-06-04

**Authors:** sathyanarayanan varadarajan

**Affiliations:** 1 SRM Medical College, SRM University

This review has been migrated. The reviewer awarded 4 stars out of 5 This interesting article is authored by a fourth-year medical student, who has brilliantly highlighted the key ways medical students can spend their time productively during lockdown. This article is full of practical tips for medical students to spend time effectively and usefully during this disastrous pandemic. He has overviewed them under three sections such as Working in isolation, Planning for the future, Mental health and well-being. These tips will be helpful for all the medical students to explore new learning strategies, to plan for their careers and future and to prioritise their mental health and the well-being of others. I greatly appreciate the author for providing effective time management strategies for his fellow students who are going to be future medical practitioners.

Reviewer Expertise:

NA

No decision status is available
